# The Sanitary Lessons of the Late War in Cuba

**Published:** 1899-07-01

**Authors:** 


### THE SANITARY LESSONS OF THE LATE WAR IN CUBA.

Addressing the American Medical Association, Dr. Sternberg, Surgeon-General in the United States Army, drew a comparison between the great Civil War and the recent war with Spain, in regard to the relation between the mortality from wounds and from disease. In both wars more men died from disease than were killed in battle or died from wounds, but the disproportion was far greater in the later than in the earlier war. During the Civil War there were 186,216 deaths from disease, and 93,969, or about half as many, deaths in battle or from wounds; while during the war with Spain 5,438 died of disease, and only 968 were killed in battle, or died of wounds, injuries, or accidents. One great cause of death in both wars was typhoid fever, which is, perhaps, unavoidable when large numbers of untrained volunteers are suddenly massed together, especially when the trained medical corps is inadequate to control the sanitary situation. What is interesting, however, in the comparison between the two periods is that in the Civil War typhoid fever not only rose rapidly when the troops were brought together, but continued to increase in prevalence for a long time afterwards, whereas in the late war, although on the first assembly of the troops typhoid rose with still greater rapidity, its prevalence very rapidly diminished when active measures were instituted for its suppression, measures among which are especially to be noted the moving of the troops into fresh camp sites, and the greater care exercised in the disposal of the excreta. Dr. Sternberg points out that the amount of typhoid fever in the camps, which was at one time very alarming, was to a large extent due to the neglect of well-known sanitary measures, and also to a very general failure on the part of medical officers attached to regiments to recognise the disease in its milder forms and during its earlier stages. The chief lesson to be learned from a study of the mortality in these campaigns is, he thinks, that it is impossible to improvise at short notice an efficient military medical service. "Physicians and surgeons from civil life," he says, "however well qualified professionally, as a rule are not prepared to assume the responsibilities of medical officers charged with administrative duties and the sanitary supervision of camps. The proper performance of such duties cannot be expected from a physician without military training or experience, no matter how distinguished a position he may have held in civil life," and he urges that an army should always possess a larger corps of trained medical officers than may appear absolutely necessary while on a peace basis, and that systematic instruction in military medicine and hygiene should be provided for the medical officers of the National Guard as well as for those of the regular army. These are lessons which we in England may well take to heart. By draining our establishment, and by the employment of civilians at our depôts, no doubt we are always able to put in the field a very smart set of medical officers for our various little wars; but all the same, in proportion to the work that would be required from it in case of real war, our Army Medical Department is lamentably short-handed, while the medical officers who figure among the volunteers are civilians pure and simple.

